# Solid-phase excitation-emission matrix spectroscopy for chemical analysis of combustion aerosols

**DOI:** 10.1371/journal.pone.0251664

**Published:** 2021-05-20

**Authors:** Gaurav Mahamuni, Jiayang He, Jay Rutherford, Byron Ockerman, Arka Majumdar, Edmund Seto, Gregory Korshin, Igor Novosselov

**Affiliations:** 1 University of Washington, Mechanical Engineering, Seattle, WA, United States of America; 2 University of Washington, Chemical Engineering, Seattle, WA United States of America; 3 University of Washington, Electrical and Computer Engineering, Seattle, WA United States of America; 4 University of Washington, Environmental and Occupational Health Sciences, Seattle, WA United States of America; 5 University of Washington, Civil and Environmental Engineering, Seattle, WA United States of America; Nazarbayev University, KAZAKHSTAN

## Abstract

Exposure to ultrafine combustion aerosols such as particulate matter (PM) from residential woodburning, forest fires, cigarette smoke, and traffic emission have been linked to adverse health outcomes. Excitation-emission matrix (EEM) spectroscopy presents a sensitive and cost-effective alternative for analysis of PM organic fraction. However, as with other analytical chemistry methods, the miniaturization is hindered by a solvent extraction step and a need for benchtop instrumentation. We present a methodology for collecting and in-situ analysis of airborne nanoparticles that eliminates labor-intensive sample preparation and miniaturizes the detection platform. Nanoparticles are electrostatically collected onto a transparent substrate coated with solid-phase (SP) solvent—polydimethylsiloxane (PDMS). The PM organic fraction is extracted into PDMS and analyzed *in-situ*, thus avoiding liquid-phase extraction. In the SP-EEM analysis, we evaluated external and internal excitation schemes. Internal excitation shows the lowest scattering interference but leads to signal masking from PDMS fluorescence for λ<250nm. The external excitation EEM spectra are dependent on the excitation light incident angle; ranges of 30–40° and 55–65° show the best results. SP-EEM spectra of woodsmoke and cigarette smoke samples are in good agreement with the EEM spectra of liquid-phase extracts. The SP-EEM technique can be used to develop wearable sensors for exposure assessments and environmental monitoring.

## 1. Introduction

Combustion-generated ultrafine particles (UFP) consist of elemental carbon (EC) and organic carbon (OC). Brown carbon (BrC), characterized by a significant fraction of OC, is a major air pollution component with known health risks [1] and adverse environmental impacts [2]. Though the severity and mortality of many diseases have been linked to UFP exposure, there is uncertainty about specific causative agents’ influence. Size distributions, particle morphologies, optical properties, and chemical compositions of these aerosols vary significantly [3,4]. The UFP chemical composition determines the potential for biochemical reactions with tissue and cells. BrC forms from the incomplete combustion of fossil, biomass, and other carbonaceous fuels. More than 100 polycyclic aromatic hydrocarbons (PAHs) cluster together to form liquid-like particles [5]. The planar nature and inherent stability of aromatic compounds are linked to the formation of soot particles [6,7]. The size of PAHs in young soot has been approximated by molecular dynamics simulations to be between four rings (Pyrene) and 19 rings (circumcoronene) [8,9]. The PAHs participating in PM formation or components condensed on PM during the secondary growth [10,11] can be oxidized in the flame [12]; however, a significant organic fraction is retained by the particle, especially in the low temperature (incomplete) combustion [13].

Total organic carbon (TOC) is an established method for estimating the PM samples’ organic fraction [14,15], Though the organic fraction in BrC contains a variety of complex hydrocarbon compounds [12,16], PAHs have been reported to be a major cause of oxidative damage [17–19]. The United States Environmental Protection Agency (EPA) has established a panel of 16 PAH compounds as priority pollutants representing a range of molecular structures with MW = 128 to 278 g/mol. More recent reports [20] suggest that larger PAH molecules have a greater carcinogenic impact [21]. Targeted PAH analysis is typically performed by laboratory methods such as GCMS and LCMS, which are not well-suited for miniaturization.

PAHs are strong candidates for analysis by spectroscopic techniques as they have high absorption coefficients and quantum yields [22]. While fluorescence spectroscopy is very sensitive (~1 ng/mL) for PAH detection [17–19], by itself, it is not specific. The UV–visible electronic transitions in sp^2^ carbon systems such as PAHs rely on π*–π transitions [23]. Studies show an inverse power law relationship between the optical band gap (OBG) and the number of benzene rings in PAHs [24,25]. An increase in MW of PAHs broadens their absorption bands towards longer wavelengths and red-shifts their emission bands due to the decreasing OBG [26,27]. This variation in spectral properties as a function of MW can be used to distinguish PAH content in combustion aerosols.

EEM introduces an orthogonal dimension to the standard fluorescence analysis by scanning over a range of single-wavelength-excitations and stacking fluorescence emissions at each excitation wavelength, which provides a three-dimensional (3D) spectral fingerprint. In this 3D matrix, fluorescence red-shifts for higher molecular weights of OC compounds. This variation allows to further differentiate the types of compounds in a complex mixture with overlapping individual spectra. EEM has been previously used for analysis of combustion PM [28–31]. The challenges of deconvolving the overlapping peaks can be addressed by data-driven techniques [13,32]. However, similar to the standard laboratory methods, EEM analysis of aerosol samples suffers labor-intensive steps involving sample collection on filters, solvent extraction, filtration, and serial dilutions, which is not amenable for miniaturized sensors. Solid-phase (SP) extraction into transparent media suitable for spectroscopic interrogation can eliminate these steps enabling in-situ analysis required to develop miniaturized sensors. PDMS provides an attractive option among solid-phase solvents as it is inert, non-toxic, non-flammable, and optically transparent. As PDMS has a relatively low fluorescent background, it is well suited for EEM analysis. This approach may be used for personal exposure assessment to combustion-generated aerosols providing information about aerosol chemical composition and exposure levels. For example, liquid phase (LP) -EEM, combined with machine learning for spectra deconvolution, has been used for source identification [32] and source apportionment of environmental samples [33]. Exposure levels can be determined based on the intensity of the signal if calibration curves are available.

In this manuscript, we demonstrate the SP-EEM technique for direct chemical analysis of combustion-generated PM. The PM is electrostatically collected onto a transparent substrate, then extracted and analyzed *in-situ*. The collection efficiency is greater than 50% for most combustion aerosols. The SP-EEM spectra for wood smoke and cigarette smoke show has excellent reproducibility and sensitivity, as demonstrated by comparing the LP-EEM spectra for wood smoke and cigarette smoke. The excitation angles were optimized to reduce scattering and reflection effects. This proof-of-concept demonstration lays a foundation for developing miniaturized sensors for exposure assessment and environmental monitoring for combustion-generated aerosols.

## 2. Experimental methods

### 2.1 Collection substrate

PDMS solution was prepared from the platinum-based Dow Corning Sylgard 184 silicone elastomer kit by mixing the base with the curing agent in a 10:1 mass ratio. Glass and quartz slides were treated with the PDMS using a spin coater (WS-650, Laurell Technologies, North Wales, PA, USA) at 2000 rpm for 1 minute. The PDMS was cured in an oven for 4 hours at 70°C. The cured PDMS layer thickness was measured to be ~150μm.

### 2.2 Particulate matter collection

**Fig 1:**
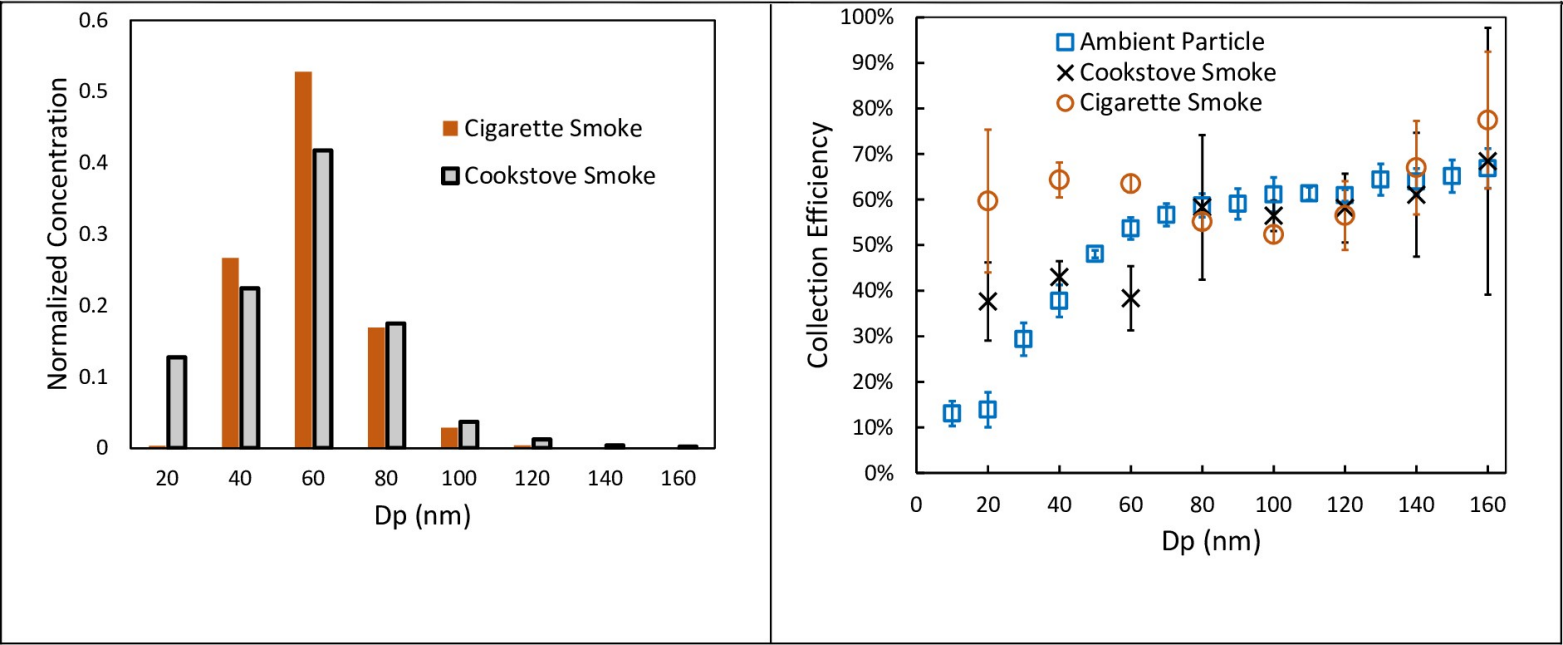
Left—Particle size distribution for Cigarette and Cookstove smokes; Right–Collection efficiency of parallel plate collector for combustion-generated aerosols and ambient particles. Collection efficiency of parallel plate collector for ambient PM as a function of particle size at 0.75 slpm. Each data point corresponds to three or more particle count measurements by a TSI SMPS NanoScan particle counter with error bars representing one standard deviation.

A miniature single-stage electrostatic precipitator (ESP) collected PM directly onto the PDMS layer, see S1 Fig in S1 File. The device did not employ a charging stage; instead, we relied on PM native charge from the combustion process. Woodsmoke was generated by burning 3.8 cm by 1.9 cm Douglas fir sticks in a side-feed natural-draft cookstove [34]. Cigarette smoke was generated by lighting cigarettes in a sealed well-mixed aerosol test chamber [35]. Size distribution was measured using a scanning mobility particle sizer (SMPS, TSI Nanoscan 3910); peak particle concentration for woodsmoke and cigarette smoke was recorded for particle diameters of dp~60 nm, see Fig 1. The collector did not employ a charging stage; instead, we relied on PM native charge from the combustion process. Two coated collection substrates were placed in a Teflon holder, 5 mm apart, forming a rectangular flow channel, with PDMS layers facing the flow. Particle-laden flow was aspirated between the collection substrates at the flow rates of 1.8 slpm. Two copper electrodes positioned on either side were connected to a high voltage power supply (Glassman High Voltage Inc. EH Series, High Bridge, NJ, USA). An electric potential φ = 3 kV was applied between the electrodes resulting in electric field strength E = 0.42 kV/mm. The collection efficiency (CE%) was determined by comparison with a parallel reference channel plumbed to SMPS [36,37], and for these conditions, CE% ~ 40–60%, see Fig 1 (right). Higher efficiency for smaller (d_p_ < 50 nm) combustion-generated was observed compared to ambient aerosol. It is likely due to the greater residual charge on the combustion particle compared to Boltzman charge distribution associated with ambient aerosol aging.

The particles were collected onto PDMS coated slide, the tests were run until visible particle deposition was observed (90 min for woodsmoke and 10min for cigarette smoke). The substrates were removed from the collector and isolated for ~ 24 hours to allow adequate time to extract organic compounds into PDMS.

The reference sample was collected for LP-EEM analysis [32,33] on PTFE membrane filters (Pall Zefluor®, Cat.# P5PJ037, Pall Corp., Port Washington, NY, USA) using Harvard impactor (Cat. # HP2518, BGI, Butler, NJ, USA). After the gravimetric analysis, the samples were extracted in cyclohexane (Cat. #1.02822.2500, Uvasol®, Millipore Sigma, Burlington, MA, USA). The extracts were filtered with 0.2 μm PTFE syringe filters (Cat. #28145–491, VWR, Edison, NJ, USA) and diluted to achieve ~3.5–5 μg/mL concentration. The filtered extracts were then analyzed by EEM using a spectrofluorometer (Aqualog-880-C, HORIBA Inc, Edison, NJ, USA). The spectra from the liquid extracts were recorded in the range of excitation wavelength *λ_ex_* = 200–600 nm with a 2 nm resolution. For each excitation wavelength, the instrument records emissions using a CCD array in the range of *λ_em_* = 246–826 nm with a 0.58 nm resolution.

The extracts were analyzed using an Agilent 7000 GC/MS Triple Quad Mass Spectrometer using two 15 m columns (Part #: HP-5MS UI, Agilent, Santa Clara, CA, USA) equipped with a backflush. 24 PAHs (EPA 16 PAHs and eight additional compounds with MW up to 302 g/mol) were included in the analysis. Calibration curves for PAH species with seven concentration levels in the range of 1–1000 ng/mL were obtained. The calibration standards for the 24 PAHs were a mixture of 23 compounds (Wellington Laboratories Cat. # PAH-STK-A, Guelph, ON, Canada) and one additional compound, coronene, a standard PAH used in several studies on mechanisms of soot formation (AccuStandard Cat. # H-116, New Haven, CT, USA). Each calibrant included 16 deuterium-labeled PAH internal standards (Wellington Laboratories Cat. # PAH-LCS-A, Guelph, ON, Canada). PAH internal standards were at a concentration of 100 ng/mL in the calibrants. An equivalent concentration of the same internal standard was spiked into each sample for use in quantification. The instrument was operated in pseudo multiple reaction monitoring (PMRM) mode. Mahamuni et al. reported the wood smoke samples’ chemical composition, calculating that the total mass of 16 EPA PAHs was ~20–25 ng/μg of soot, see S6 Fig in S1 File. About 75% (by weight) of PAHs in the sample were high species with MW>202 g/mol (i.e., larger than Pyrene) [13]. TOC analysis was not performed here but typically reported TOC values for cookstoves are greater than 50% depending on the type of the cookstove and operational conditions [38–40]. The smoldering combustion during cigarette smoking does create any elemental carbon, and generated particles remain in the organic phase as the carbonization threshold is not reached [11,41].

### 2.3 Solid-phase excitation emission matrix analysis

**Fig 2:**
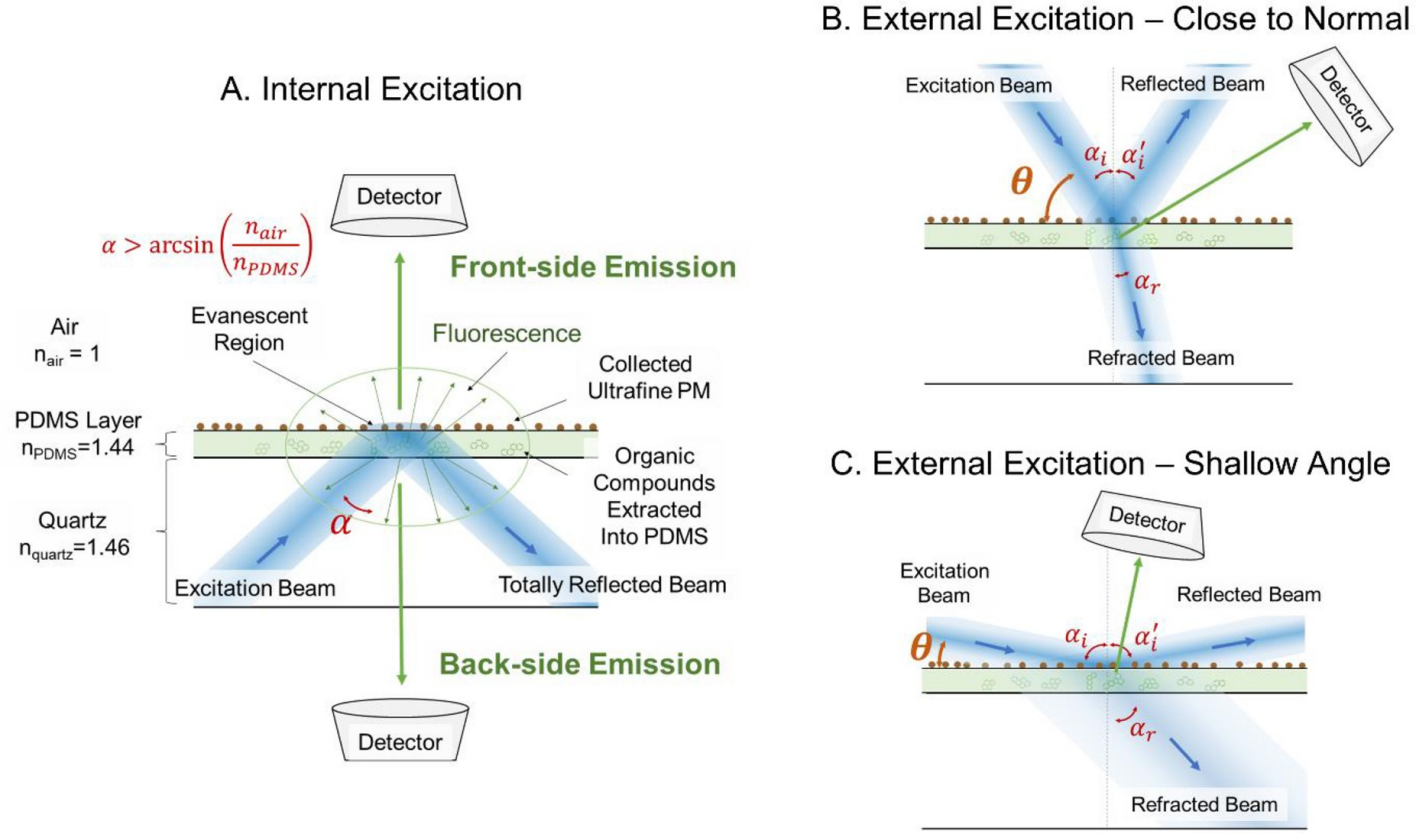
(A) In internal excitation, light passes through the analysis substrate, and the fluorescence signal is recorded from the PDMS side of collection substrate (front-side emission, θ = 0°) or the uncoated side of the collection substrate (back-side emission, θ = 180°). (B) SP-EEM analysis using external excitation for excitation angle (θ) closer to a normal and (C) SP-EEM analysis using external excitation for shallow excitation angle between the collection surface and excitation light; the angle between the incident light and collection optics was held constant at 90°.

The SP-EEM spectra of PM collected on the analysis substrates were obtained using Aqualog-880-C with a microscope slide holder. The angle between the incident excitation light and the substrate (θ) was varied from 5° to 85° to optimize for maximum fluorescence signal and minimum Rayleigh and Raman scattering interference, see Fig 2B and 2C. Elastic scattering and reflection intensities are significantly greater in SP-EEM compared to LP-EEM measurements, due to the presence of PM and coating surface non-uniformity (in LP-EEM, the sample was filtered). SP-EEM measurements taken at 5°<θ<85° are referred to as external excitation. In the external excitation, both glass and quartz substrates produce similar results; see SI Section 3.

Internal excitation was explored for θ = 0° and θ = 180°, there the quartz substrate acts as a waveguide for excitation light. It has been shown previously that in internal excitation, interference of scattering signal with the fluorescence is lower compared to external excitation [36]. Since transmission of glass is negligible for λ<300nm (see S4 Fig in S1 File), quartz substrates were used in internal excitation experiments. Excitation light couples to transparent PDMS layer due to refraction or by evanescent wave mechanism. The excitation light interacts with organic compounds extracted into the PDMS layer. The excited compounds emit fluorescence, which is collected at a 90° angle to the excitation beam, see Fig 2A. We refer to θ = 0° configuration as ’front-side emission’ as fluorescence emission is collected from the PM deposition side of the substrate, while θ = 180° configuration is referred to as ’back-side emission’ in which fluorescence emission is collected from the side opposite to the PM deposition. SP-EEM of a blank substrate was subtracted from each PM sample. Rayleigh and Raman scattering peaks were removed computationally [42]. The processed EEMs were passed through a Gaussian filter (σ = 2) and the negative values were removed numerically (MATLAB, MathWorks Inc., Natick, MA, USA). The resulting EEMs were normalized to Raman units (R.U.).

## 3. Results and discussion

The external excitation experiments with woodsmoke samples focused on optimizing the excitation angle. The fluorescent peak intensity was observed to increase to about θ = 50° and then decreased (S2 Fig in S1 File). The combined reflection and scattering signals are at their maximum at θ = 40°-55° masking the fluorescent signal. Low excitation angles θ = 5–20° did not result in a sufficiently strong fluorescent signal likely due to the broadening and diffusion of the excitation beam in the substrate lowering excitation intensity, see Fig 2C. At angles θ >70°, the fluorescent signal drops significantly likely due to the decrease in the excitation path length and reduction of the view factor of the collection optics, as shown in Fig 2B. Additional ray optics modeling is required to gain a better understanding of the system. Two angle ranges were found as optimal by balancing the fluorescent spectra (signal) and scattering/reflection intensity (noise); these are θ = 30–40° and θ = 55–65°. For the comparison with other samples and methods, θ = 60° is used hereafter.

**Fig 3:**
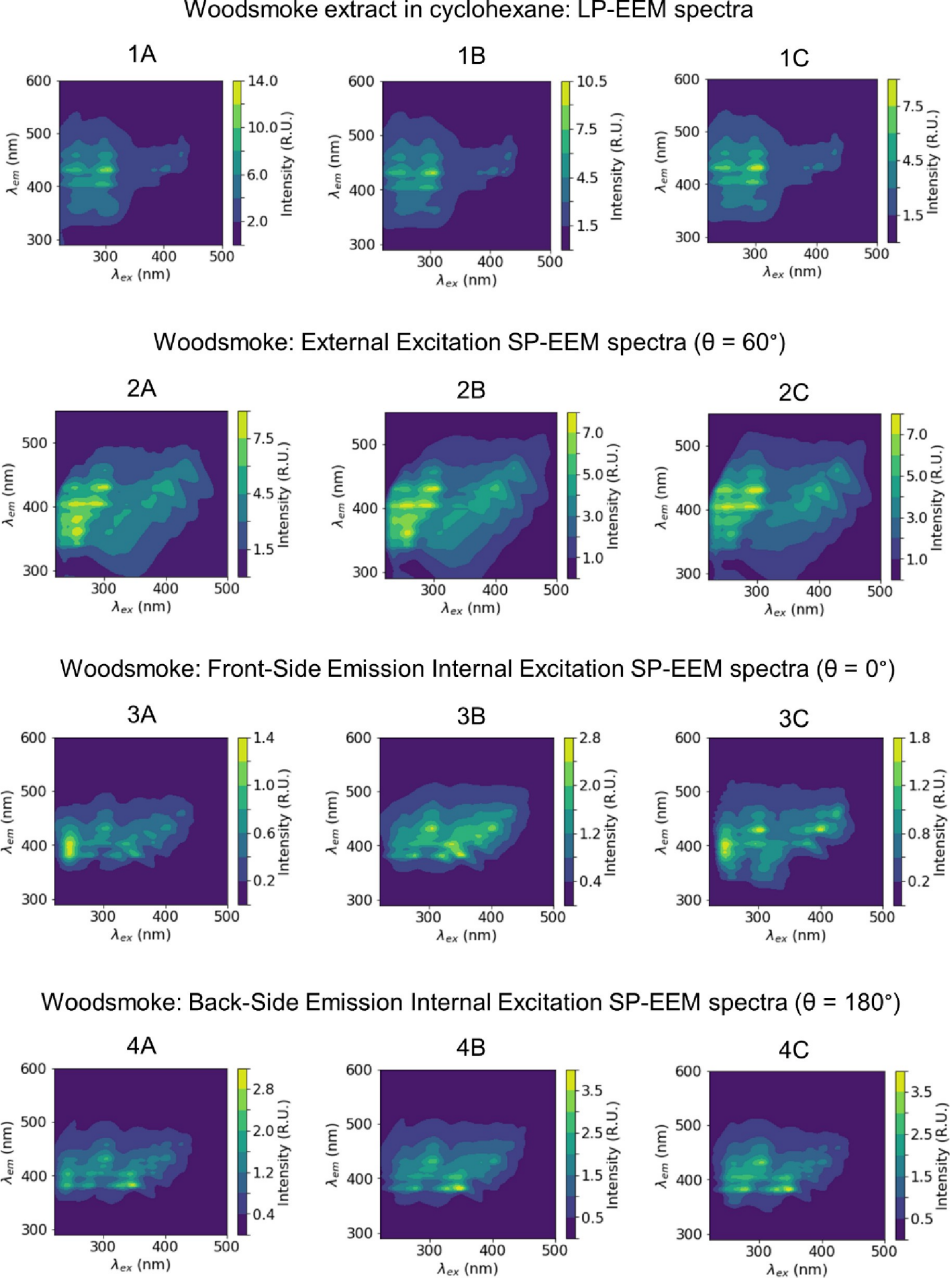
Comparison of SP-EEMs obtained using three different excitation configurations and LP-EEMs for woodsmoke PM. For each optical configuration, three samples (A, B, C) were analyzed. The external excitation SP-EEMs match well with LP-EEMs. Both front-side emission and back-side emission internal excitation SP-EEMs lose spectral information for λ_ex_ < 250nm due to interference of PDMS fluorescence (S5 Fig in S1 File).

The internal excitation SP-EEMs generated at θ = 0°, θ = 180°, these are compared to external excitation θ = 60° for three different woodsmoke samples in Fig 3. In the internal excitation arrangement, the PDMS layer acts as a waveguide. As a result, the optical path length is significantly greater than that in external excitation, resulting in a higher PDMS fluorescent background (S5 Fig in S1 File). Blank signal subtraction results in either (i) the masking of sample fluorescence peaks at λ_ex_< 250nm as visible in Fig 3 (3A and 3C) or (ii) the suppression or complete removal of woodsmoke fluorescence peaks at λ_ex_< 250nm as shown in Figs 3 (3B, 4A–4C). In the internal excitation SP-EEMs, the scattering peaks’ intensity is lower compared to those seen in the case of external excitation. After subtracting the background scatter and interpolation of fluorescence [32,33], the internal excitation yields better-resolved features near the scattering lines than in the external excitation arrangement. This is beneficial for analyzing the samples with high molecular weight (more complex) compounds as their EEM fingerprints are typically shifted to the upper-right corner of the spectra.[43].

**Fig 4:**
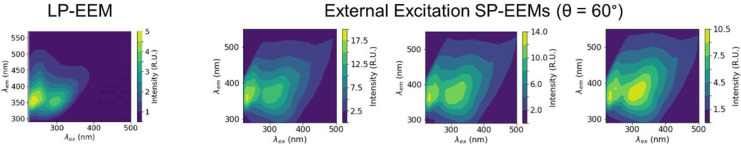
External excitation SP-EEMs for cigarette smoke show that most of the peaks agree with LP-EEM. The removal and interpolation of Rayleigh and Raman scattering lines lead to broadening or removing fluorescence in SP-EEMs compared to LP-EEMs, similar to woodsmoke signatures (Fig 3).

To demonstrate the applicability of the SP-EEM to samples other than wood smoke, external excitation SP-EEM analysis (at θ = 60°) was performed for cigarette smoke. Fig 4 shows that the SP-EEMs for these samples have excellent repeatability and correlation with LP-EEM. Cyclohexane extraction yielded spectra with higher relative peak intensity for λ_ex_ < 250nm, which is possible due to differences in extraction efficiencies or uncertainties in the background subtraction (i.e., PDMS fluorescence, see S5 Fig in S1 File) in the SP-EEM analysis. Extraction efficiencies depend on the solvent; Rutherford showed that EEM spectra for methanol and cyclohexane were similar, while water extracts showed different spectra [32]. If cyclohexane is more efficient at extracting lower MW compounds than PDMS, the SP-EEM peak associated with these compounds would be suppressed.

The spectra were obtained from two combustion sources, i.e., cigarette smoke and wood smoke, and by three SP-EEM devices. The reference filter was used to collect the sample in parallel and was analyzed by LP-EEM. The fluorescent peak locations between the SP-EEM measurements for each source are identical, showing good reproducibility of the analysis method. The of the EEM spectra intensities are within 10% for all SP-EEMs collected in parallel, suggesting that (i) the collection efficiency for the devices are similar and (ii) extraction and analysis approach are reproducible. The high intensity of the peaks and the spectra resolution will allow for the method’s usability for source apportionment studies [32,33], and PM PAH classification based on their molecular weight [43,44].

## 4. Conclusions

This manuscript presents a novel approach to a chemical analysis of combustion-generated aerosols. This approach eliminates the need for extraction into the liquid phase; the measurements can be performed in-situ, providing the foundation for sensor development. Optical configuration and the choice of the solid phase solvent should be considered in the optimization. In external excitation, for shallow angles, excitation intensity is lower; for angles close to normal, one needs to consider optical path length, the solid angle of the detection optics, and losses due to the inner filter effect [45]. Internal excitation results in stronger interference from PDMS fluorescence for λ_ex_ < 250nm. For compounds that fluorescence at longer wavelengths, such as higher molecular weight hydrocarbons, the internal excitation can provide significant advantages in sensor design, reducing elastic scatter and reflections from the excitation light source. While the feasibility of combustion source identification based on spectral features is demonstrated, further development of EEM libraries is required. The usability of the approach for mixed sources and complex environmental samples needs to be further investigated. Our previous work successfully classified cigarette, diesel, and woodsmoke sources as present or absent in a series of laboratory samples with overall accuracy for all three sources at 89% [32]. We expect similar results from the SP-EEM approach. Exposure level can be determined based on the intensity of the signal with proper calibration of the method. Limits of detection and saturation need to be considered in future work. Other solid-phase solvents with lower fluorescent signatures can be used to reduce fluorescent background.

## Supporting information

S1 File (DOCX)

S1 Graphical abstract (DOCX)
