# Supplementary material for: Solid-phase excitation-emission matrix spectroscopy for chemical analysis of combustion aerosols
Source: PLoS One. 2021 May 20;16(5):e0251664. doi: 10.1371/journal.pone.0251664 (PMC8136721; doi:10.1371/journal.pone.0251664)
Supplement: S1 File — (DOCX) [file pone.0251664.s001.docx]

**Solid Phase Excitation-Emission Matrix Spectroscopy for Combustion Generated Particulate Matter Analysis**

*Gaurav Mahamuni^1^, Jiayang He^1^, Jay Rutherford^2^, Byron Ockerman^1^, Arka Majumdar^3^, Edmund Seto^4^, Gregory Korshin^5^, Igor Novosselov*^,1^*

^1^ University of Washington, Mechanical Engineering, Seattle, WA 98195

^2^ University of Washington, Chemical Engineering, Seattle, WA 98195

^3^ University of Washington, Electrical and Computer Engineering, Seattle, WA 98195

^4^University of Washington, Environmental and Occupational Health Sciences, Seattle, WA 98195

^5^ University of Washington, Civil and Environmental Engineering, Seattle, WA 98195

**Supplemental Information**

Number of Pages: 8

Number of Figures: 9

# Particulate Matter Collection

| 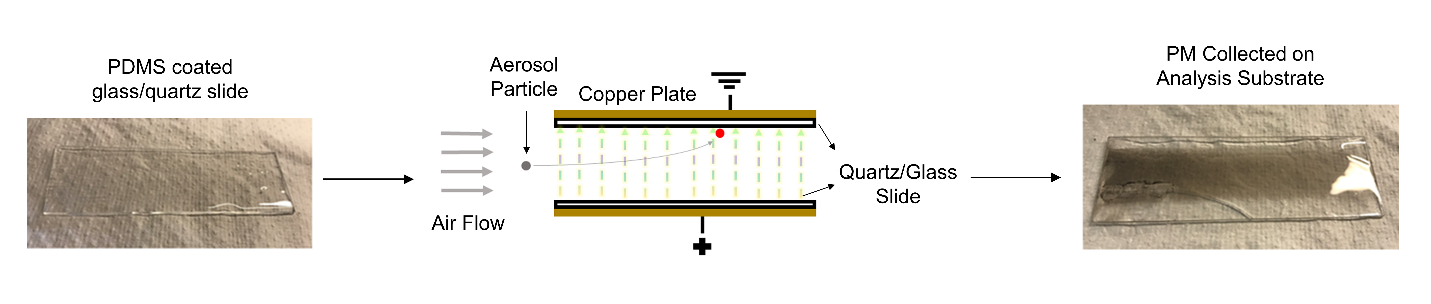 |
| --- |
| Figure S1: Quartz slide coated with PDMS (left) was placed in a parallel plate electrostatic collector. Combustion-generated PM was collected directly onto the PDMS coated surface of the substrate. The substrate was used for SP-EEM analysis after collection. |

# External Excitation SP-EEM Measurement

External excitation SP-EEMs of woodsmoke at varying θ are plotted in S2 without removing the Rayleigh and Raman scattering peaks computationally. As θ increases from 0° to 50°, the intensity of woodsmoke fluorescence peaks increases; however, a greater EEM region is masked due to wider Rayleigh and Raman scattering peaks. The same is observed when θ decreases from 85° to 50°. At θ = 50°, the intensity of woodsmoke fluorescence peaks is the highest among all SP-EEMs, however, the scattering peaks block almost all the fluorescence peaks except in the region λ_ex_ = 280nm – 320nm, λ_em_ = 380nm – 480nm. These masked external excitation SP-EEM features cannot be recovered using interpolation of scattering peaks as in the LP-EEMs because the scattering peaks are significantly broader compared to the fluorescence peaks. For θ = 0° or θ = 85°, the scattering peaks are narrower, the intensity of the fluorescence signal is too low.

For smaller θ, most of the light detected is reflection/scattering. As θ increases, the path of reflected/scattered light moves away from the detector, and PM fluorescence peaks are observed in SP-EEMs. For θ > 50°, as θ increases, the path of emitted fluorescence light through PDMS increases. This reduces PM fluorescence reaching the detector due to the inner filter effect.^1^ To balance the increasing intensity and masking of fluorescence, θ = 60° was chosen as optimum θ since, at θ = 60°, removal of scattering peaks computationally retains fluorescence peaks defining the EEM signature of woodsmoke with good intensity levels, as shown in Figure S2.

| 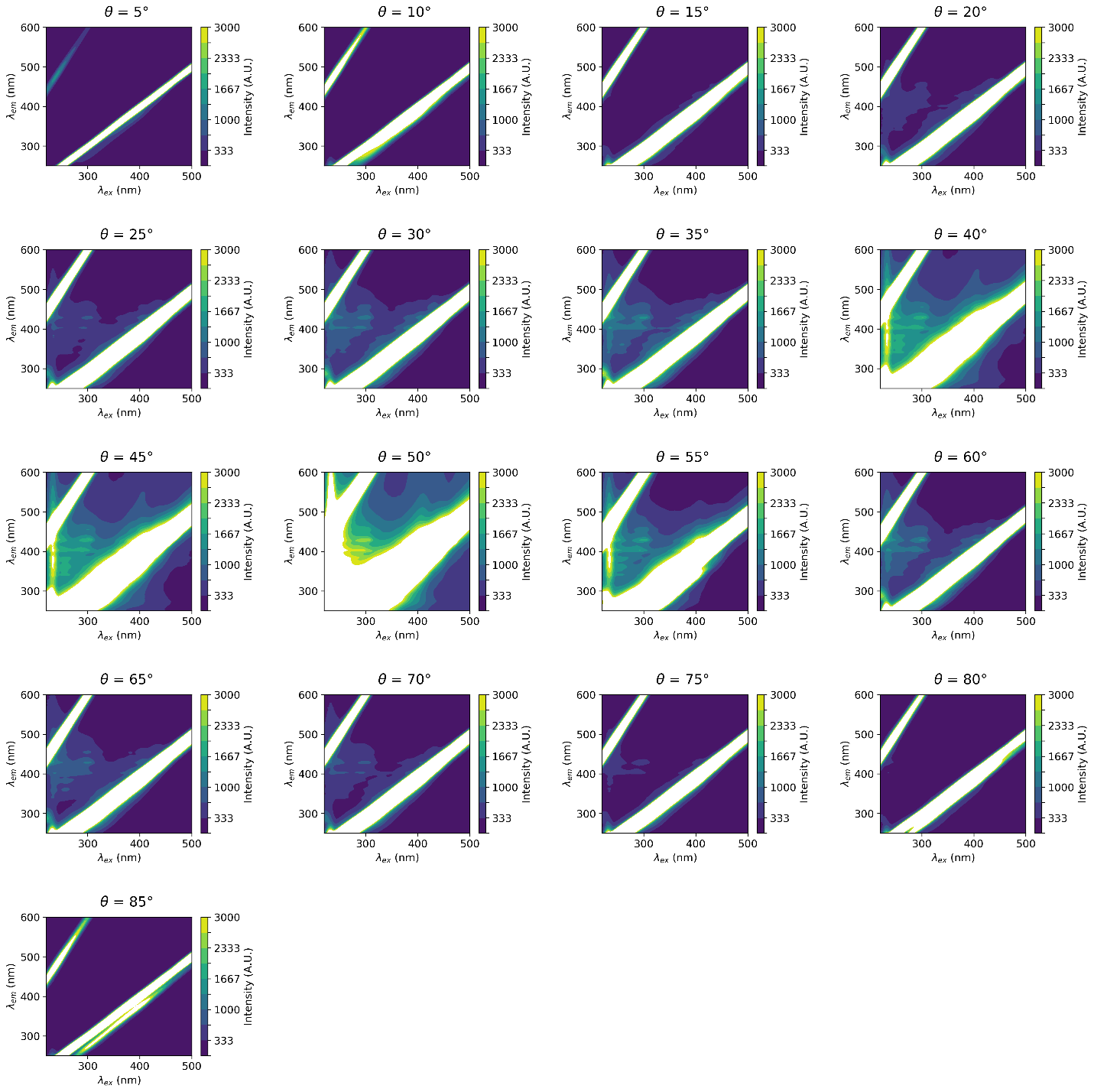 |
| --- |
| Figure S2: External excitation SP-EEMs of woodsmoke at varying θ. θ = 60° was chosen as an optimum angle for external excitation SP-EEM measurements. |

# External Excitation SP-EEM Comparison of Quartz and Glass Analysis Substrates

Figure S3 shows external excitation SP-EEMs at θ = 60° recorded using glass and quartz analysis substrates. The number and relative intensity of peaks in the woodsmoke PM fluorescence signature matches well in both SP-EEMs (see Figure S3), and it does not affect external excitation SP-EEM measurements. However, this difference in transmission will affect internal excitation SP-EEM measurements where the substrate acts as a waveguide for excitation light, in which case glass analysis substrates cannot be used due to negligible transmission of UV light (λ < 300nm). Hence, we use quartz analysis substrates for internal excitation SP-EEM measurements in the main manuscript.

| \| Glass Analysis Substrate \| Quartz Analysis Substrate \| \| --- \| --- \| \| 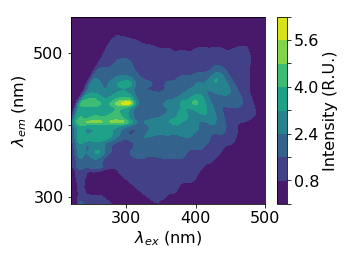 \| 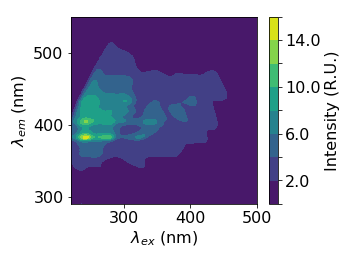 \| |
| --- | --- | --- | --- | --- |
| Figure S3: External excitation SP-EEMs at θ = 60° match well for glass and quartz analysis substrates. The number and relative intensity of peaks defining the woodsmoke PM fluorescence signature is similar for both SP-EEMs. |

# Transmission Spectra for Quartz and Glass

| 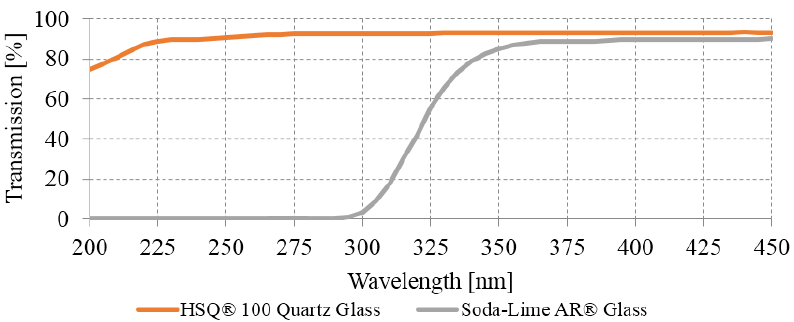 |
| --- |
| Figure S4: Transmission of HSQ® 100 quartz glass (92% at 280 nm) and soda-lime glass (2% at 280 nm) with a thickness of 1 mm between 200 and 450 nm measured with a Specord 250 Plus spectrophotometer from Analytik Jena (Jena, Germany).^2^ |

# Interference of PDMS Fluorescence in Internal vs. External Excitation SP-EEMs

Figure S5 shows external excitation SP-EEM at optimum θ (θ = 60°), front-side emission, and back-side emission internal excitation SP-EEMs of woodsmoke PM samples without subtracting SP-EEM of a blank analysis substrate during pre-processing of EEM data. PDMS fluorescence (λ_ex_ < 250nm, 350nm < λ_em_ < 450nm) overlaps with woodsmoke PM fluorescence. For external excitation SP-EEM, the PDMS fluorescence is negligible compared to woodsmoke PM fluorescence.

| 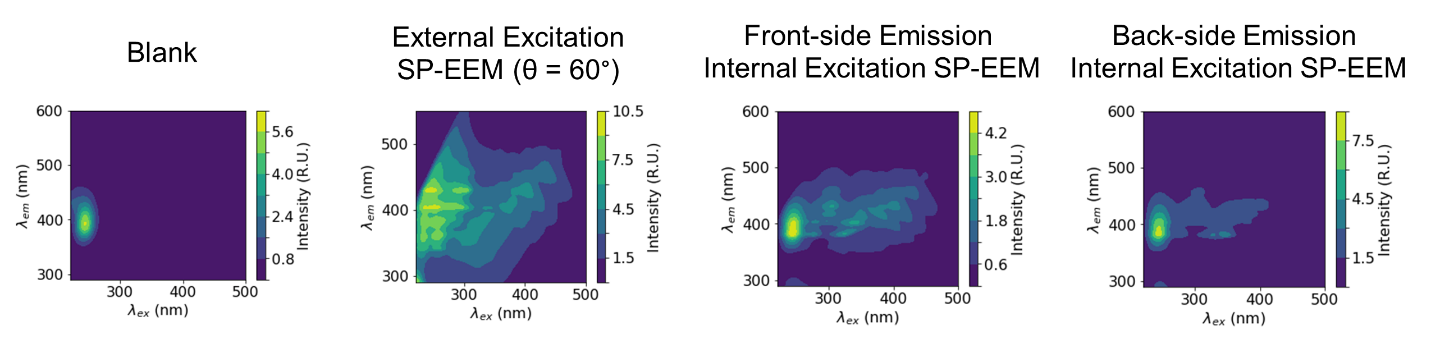 | | |  |
| --- | --- | --- | --- |
| Figure S5: SP-EEMs of woodsmoke PM at θ = 60° without blank subtraction using three different excitation emission optics arrangements. Intensity of PDMS fluorescence (λ_ex_ < 250nm, 350nm < λ_em_ < 450nm) compared to woodsmoke fluorescence peaks (λ_ex_  > 250nm) is least in external excitation SP-EEM. | | |  |
| 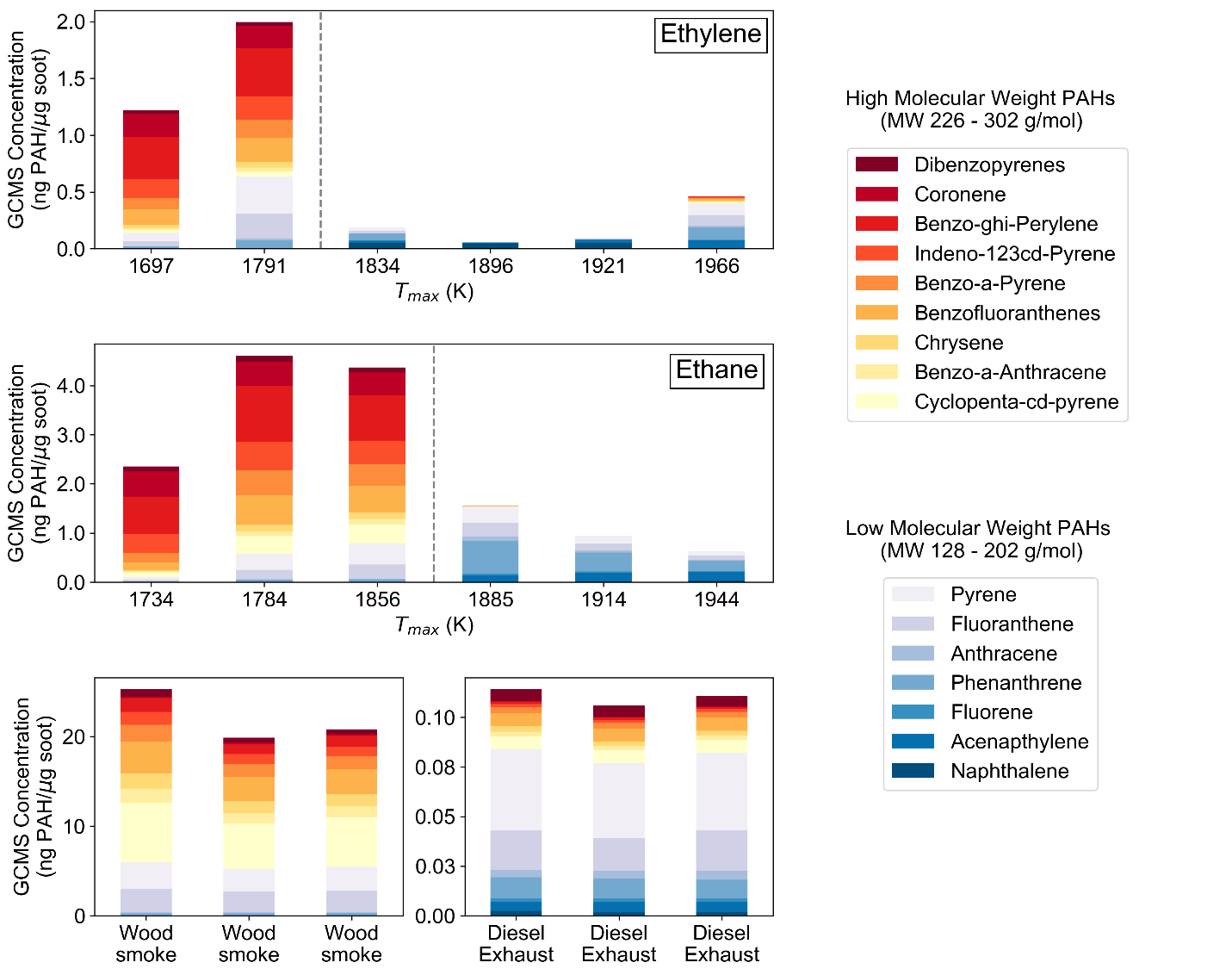 | 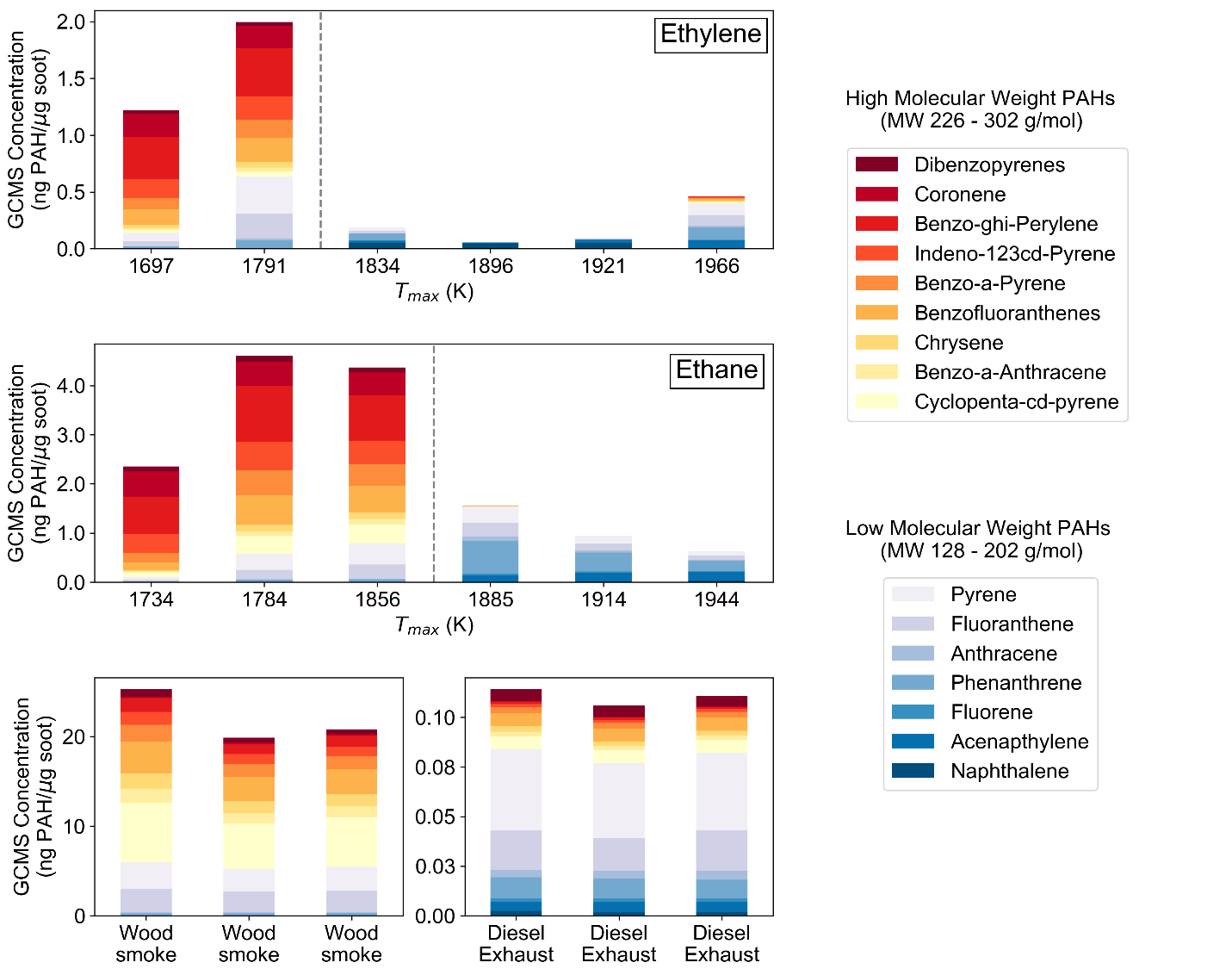 | 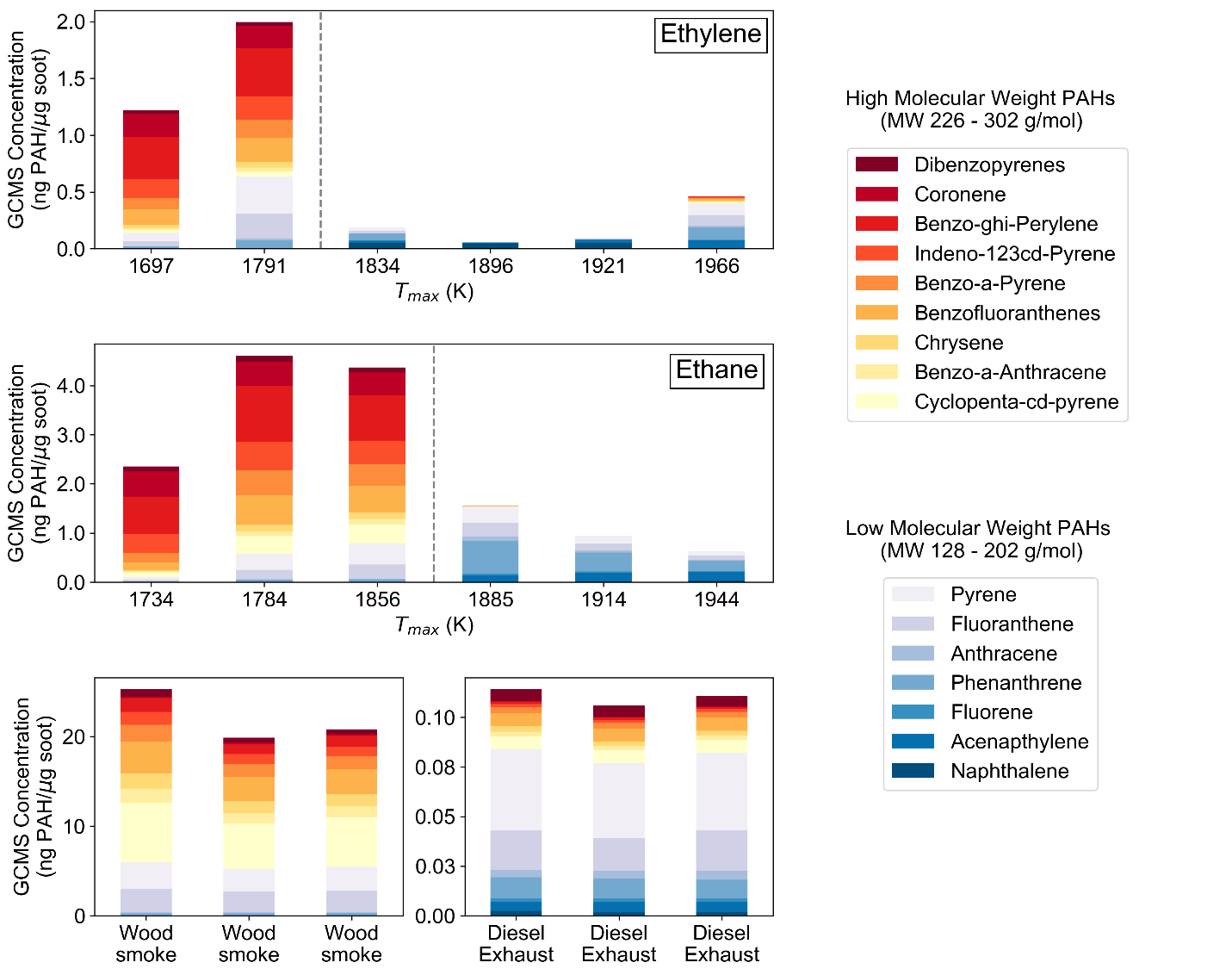 | |

Figure S6: PM PAH fraction for woodsmoke. GCMS concentration of 16 PAHs is divided into LMW and HMW PAH. The woodsmoke contains mainly HMW PAHs.

# References

(1) Larsson, T.; Wedborg, M.; Turner, D. Correction of Inner-Filter Effect in Fluorescence Excitation-Emission Matrix Spectrometry Using Raman Scatter. *Anal. Chim. Acta* **2007**, *583* (2), 357–363. https://doi.org/10.1016/j.aca.2006.09.067.

(2) Gross, A.; Stangl, F.; Hoenes, K.; Sift, M.; Hessling, M. Improved Drinking Water Disinfection with UVC-LEDs for Escherichia Coli and Bacillus Subtilis Utilizing Quartz Tubes as Light Guide. *Water* **2015**, *7* (9), 4605–4621. https://doi.org/10.3390/w7094605.
