# Supplementary figures and images for: Solid-phase excitation-emission matrix spectroscopy for chemical analysis of combustion aerosols
Source: PLoS One. 2021 May 20;16(5):e0251664. doi: 10.1371/journal.pone.0251664 (PMC8136721; doi:10.1371/journal.pone.0251664)

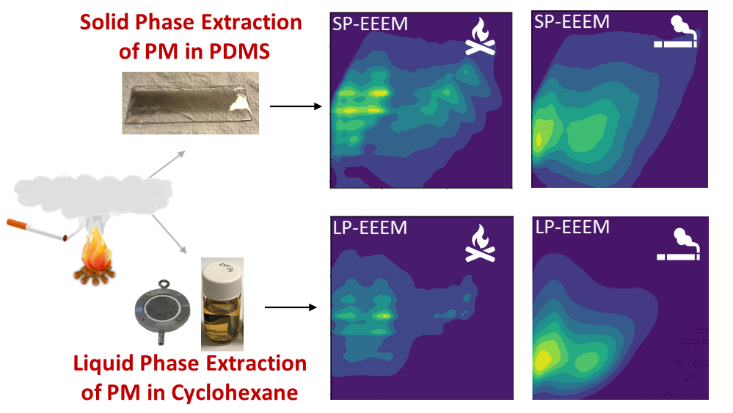

Supplement: S1 Graphical abstract — (DOCX) [file pone.0251664.s002.docx]
